# Editorial: Expert Opinions in Network bioinformatics: 2022

**DOI:** 10.3389/fbinf.2023.1254668

**Published:** 2023-07-19

**Authors:** Paola Lecca

**Affiliations:** Faculty of Engineering, Free University of Bozen-Bolzano, Bolzano, Italy

**Keywords:** systems biology, biological network, network medicine, computational biology, bioinformatics

Network biology is often referred to as systems biology. Systems biology is a fresh interpretation of established ideas (Voit, 2022). Several researchers started to develop methods that would allow representing and analyzing nonlinear models of, in theory, arbitrarily complex systems. These methods embraced Claude Bernard’s early ideas of control processes governing life (Bernard, 1865; Noble, 2007), and more specifically the tenets of dynamical systems analysis, as elegantly proposed by Ludwig von Bertalanffy (von Bertalanffy, 1927; von Bertalanffy, 1968; von Bertalanffy, 1940; Drack et al., 2007). Starting from this basis, a few scholars have begun to create methods for describing and analyzing nonlinear models with theoretically any amount of complexity. The phrase “systems biology” was first used in relation to a symposium in 1968 by Mesarović (Systems Symposium and Mesarović, Mihajlo D, and Case Institute of Technology and Mesarovic, 1968). As Voit states in a recent perspective article that also traces the birth of systems biology (Voit, 2022), the insiders had begun to realize that computational methods were required to fully realize the concepts and promise of systems biology. These methods would have to specifically deal with biological phenomena as complex, adaptive, and dynamic systems. In order to support the functional integration of small models (or “modules”) into progressively larger, well-organized ensembles of models in complex environments, these methods have to be able to deal with time evolution and complex non-linearities. This would have required the representation of a system as a network (modeled as a graph or hypergraph) and the description of its static and dynamic properties through mathematical concepts, which would later become the foundation of the algorithms simulating the time evolution of such a complex system at different scales (Ma’ayan, 2011; Yan et al., 2017).

Systems biology today is defined as the holistic study (in opposition to the reductionist approach) of complex systems in which omics methods such as transcriptomics, glycomics, and lipidomics are widely used. Systems biology seeks to comprehend, with the help of computer-simulated numerical and mathematical model simulations, the network of connections and the effects of these connections on a system scale, involving even hundreds of distinct biological molecules at the same time. Then, slowly, the concept of system-level conceptualization of a set of processes, phenomena, and data was extended from biology to medicine, creating what is known as network medicine (Milano and Cannataro, 2023). In the bosom of systems biology and systems medicine, nurtured by systems theory and computer science, network bioinformatics was born.

Voit (2022) identified four aspects which drove a gradual shift from the reductionist approach to the system-level approach in biology and consequently in computational biology. The first two were the impending completion of the entire human genome project and the burgeoning and rapidly expanding availability of high-throughput technologies that created unprecedentedly huge amounts of molecular data (Altaf-Ul-Amin et al., 2014). The third was the growth in computer power, which made it available to nearly anybody on the planet. Finally, influential scholars such as Leroy Hood (Ideker et al., 2001; Hood et al., 2004) and Hiroaki Kitano (Kitano, 2002) were able to persuade the community of the importance of systems biology and of the shift from mathematical biology to computational biology and bioinformatics that begun as a new field of science around 2000.

Twenty years have now passed since the inception of network bioinformatics, but the interest in the field and the continuing contributions that this discipline provides in support of both theoretical and applied research in biology, medicine, pharmacology, epidemiology, clinics, healthcare, and even ecology are now indispensable. This is the reason for still talking about network bioinformatics today so that its role and the commitment of so many scholars and experts in the field are not taken for granted and the developments of this discipline can be continually monitored both from a technical-scientific as well as an ethical and philosophical point of view. This is the reason for this Research Topic.

The Research Topic hosts five articles: three Original Research Papers, one Review, and one Perspective article. Original Research articles include.

• Inference of Dynamic Interaction Networks: A Comparison Between Lotka-Volterra and Multivariate Autoregressive Models by Olivença et al., where the authors address the problem of inferring a network from experimental data. The authors pointed out that the inference of biological networks from data still presents a difficulty that is mostly unaddressed despite significant efforts by the scientific community. The idea that interactions between molecular or organismal populations are static and correlative is a common approach to addressing the structure of networks. The authors claim that, while frequently effective, these techniques are not a cure-all. When the network nodes represent quantities that are dynamically changing, conclusions become more difficult because these techniques typically neglect the asymmetry of interactions between two species. To address these issues, two very distinct network inference approaches have been proposed in literature: the Lotka-Volterra (LV) models and Multivariate Autoregressive (MAR) models. Olivença et al. compared these dynamic network inference techniques side by side while analyzing datasets that were both artificially created and obtained from the natural world. MAR and LV models are mathematically equivalent at the steady state, but the results of the comparison suggested that LV models are generally better at capturing the dynamics of networks with non-linear dynamics, whereas MAR models are better suited for analyses of networks of populations with process noise and close-to-linear behavior.
• Prediction of adverse drug reaction linked to protein targets using network-based information and machine-learning by Galletti et al. In this study, the authors dealt with the problem of predicting the unpleasant side effects of drugs. Adverse drug reactions (ADRs) brought on by the modification of specific protein targets might aid in the development of safer medications, hence lowering the financial losses brought on by high attrition rates. The authors suggested a target-centric strategy to forecast relationships between protein targets and ADRs as an alternative to the more conventional drug-centric strategy. A machine-learning classifier that incorporates a collection of eight distinct network-based features serves as the foundation for the predictor’s implementation. These comprise a set of network descriptors in the form of degree and betweenness centrality measurements, conservation, a network distance to proteins that are part of safety panels used in preclinical drug development, a network diffusion-based score, the identification of protein modules based on network clustering algorithms, functional similarity among proteins, and network distance to proteins that are part of safety panels used in preclinical drug development. A variety of machine learning classifiers were utilized in the study to construct predictors using this heterogeneous set of characteristics. The level of accuracy of the predictors, according to the authors, justifies their use for the identification of protein targets that are troublesome, both at the level of a single ADR and a collection of connected ADRs arranged according to common system organ classes. The authors used the prediction of ventricular tachycardia as an example, which had a 0.70 Matthew Correlation Coefficient and accuracy and precision of 0.83 and 0.90, respectively.
• Investigating the molecular mechanism of Iguratimod act on SLE using network pharmacology and molecular docking analysis by Zeng et al., where the authors applied network pharmacology approaches to investigate the pharmacological mechanisms of Iguratimod, a novel small disease-modifying compound widely used in Asia for the treatment of rheumatic diseases such as the Systemic Lupus Erythematosus (SLE). IGU’s probable active molecules were screened using the UNIPRO and OMIM databases, and prospective targets for the compound’s actions were predicted. Using the PPI network created by the String database, the hub targets genes at the intersections of the possible targets (IGU), and associated genes (SLE) were validated. In this study, the aforementioned database was searched for 292 possible IGU targets, 6501 SLE-related illness targets, and 114 cross targets. Ten hub targets were found by network topology analysis, including CASP3, AKT1, EGFR, MMP9, and IGF1. The negative control of the apoptotic process and signal transduction are the core topics of GO enrichment analysis. The PI3K-AKT signaling pathway, MAPK signaling pathway, and FoxO signaling pathway may be important players in the pharmacological mechanisms through which IGU affects SLE, according to the KEGG enrichment study. The IGU ligand demonstrated high binding activity to the hub targets, according to molecular docking.

The Review includes the following:

1. A brief survey on tools for genomic regions enrichment analysis, by Chicco and Jurman. This is a Mini Review reporting the state of the art on the tools for genomic regions enrichment analysis. In particular, the authors review and compare six tools: BEHST, g:Profiler g:GOSt, GREAT, LOLA, Poly-Enrich, and ReactomePA. The results of the comparison show that these technologies frequently include data from regulatory components, like ChIP-seq, which is thought to enhance the outcomes of the enrichment analysis.

The Perspective includes the following:

1. Network medicine: Facilitating a new view on Complex Diseases by Cvijovic and Polster. This article is about understanding complex diseases through approaches of network medicine. According to the authors, complex diseases are difficult and challenging to study, comprehend, prevent, and treat because they include various levels of biological organization in the context of environmental and psychosocial factors. The study of network medicine has advanced the comprehension of these intricate systems and revealed patterns of symptom co-occurrence as well as the mechanistic overlap between diseases. The authors say that these findings force us to reconsider our nosological models and challenge the conventional view of complicated disorders, which treat diagnoses as separate entities. Therefore, Cvijovic and Polster proposed a novel paradigm in which the state vector of the individual disease burden, which simultaneously depends on molecular, physiological, and pathological parameters, is provided. This paradigm switches the emphasis from understanding the underlying pathophysiology of diagnosis cohorts to analyzing the characteristics of each patient’s symptom. In the context of complicated disorders, this perspective makes it easier to take a multifaceted approach to understanding human physiology and pathology. In order to advance toward personalized therapy, this may offer a valuable notion to handle the high inter-individual variation of diagnostic cohorts as well as the haziness of the difference between diagnosis, health, and disease.

In summary, with these contributions, this Research Topic would like to draw the readers’ attention to some interesting new research developments in the field of network bioinformatics, in particular, the still largely unresolved problem of inferring the network structure of interactions from experimental observations, the determination of adverse drug effects from the analysis of causal drug-target networks, the functional enrichment of gene sets and thus of networks, and to the mechanisms underlying complex diseases and health disorders. Each paper, although classified here in the areas of research, review, and perspective paper, provides insights and perspectives for future research and indicates the next directions in network bioinformatics in light of the increasingly complex reality in which biological and medical science is placed and also in the light of new Artificial Intelligence techniques and methodologies.
